# Tris(ethyl­enediamine-κ^2^ *N*,*N*′)nickel(II) naphthalene-2,7-disulfonate

**DOI:** 10.1107/S1600536811049063

**Published:** 2011-11-25

**Authors:** Shan Gao, Seik Weng Ng

**Affiliations:** a Key Laboratory of Functional Inorganic Material Chemistry, Ministry of Education, Heilongjiang University, Harbin 150080, People’s Republic of China; b Department of Chemistry, University of Malaya, 50603 Kuala Lumpur, Malaysia; c Chemistry Department, Faculty of Science, King Abdulaziz University, PO Box 80203 Jeddah, Saudi Arabia

## Abstract

The Ni^II^ atom in the title salt, [Ni(C_2_H_8_N_2_)_3_](C_10_H_6_O_6_S_2_), is chelated by three ethyl­enediamine ligands in an octa­hedral geometry. The cation and anion are linked by N—H⋯O hydrogen bonds into a three-dimensional network. One of the two –SO_3_ groups is disordered over two positions in a 1:1 ratio.

## Related literature

For the structure of tris­(ethyl­enediamine)­nickel(II) 2,6-naph­thalene­disulfonate monohydrate, see: Huo *et al.* (2004 ▶).
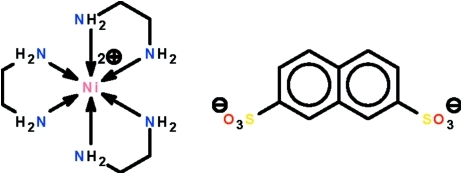


## Experimental

### 

#### Crystal data

[Ni(C_2_H_8_N_2_)_3_](C_10_H_6_O_6_S_2_)
*M* *_r_* = 525.59
Monoclinic, 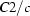

*a* = 23.624 (8) Å
*b* = 14.203 (6) Å
*c* = 14.715 (4) Å
β = 115.152 (12)°
*V* = 4469 (3) Å^3^
*Z* = 8
Mo *K*α radiation
μ = 1.10 mm^−1^
*T* = 293 K
0.20 × 0.16 × 0.13 mm

#### Data collection

Rigaku R-AXIS RAPID IP diffractometer
Absorption correction: multi-scan (*ABSCOR*; Higashi, 1995 ▶) *T*_min_ = 0.810, *T*_max_ = 0.870
21605 measured reflections
5107 independent reflections
4533 reflections with *I* > 2σ(*I*)
*R*_int_ = 0.015

#### Refinement

*R*[*F* ^2^ > 2σ(*F* ^2^)] = 0.031
*wR*(*F* ^2^) = 0.084
*S* = 1.05
5107 reflections
337 parameters
36 restraints
H atoms treated by a mixture of independent and constrained refinement
Δρ_max_ = 0.57 e Å^−3^
Δρ_min_ = −0.36 e Å^−3^

### 

Data collection: *RAPID-AUTO* (Rigaku, 1998 ▶); cell refinement: *RAPID-AUTO*; data reduction: *CrystalClear* (Rigaku/MSC, 2002 ▶); program(s) used to solve structure: *SHELXS97* (Sheldrick, 2008 ▶); program(s) used to refine structure: *SHELXL97* (Sheldrick, 2008 ▶); molecular graphics: *X-SEED* (Barbour, 2001 ▶); software used to prepare material for publication: *publCIF* (Westrip, 2010 ▶).

## Supplementary Material

Crystal structure: contains datablock(s) global, I. DOI: 10.1107/S1600536811049063/nk2123sup1.cif

Structure factors: contains datablock(s) I. DOI: 10.1107/S1600536811049063/nk2123Isup2.hkl

Additional supplementary materials:  crystallographic information; 3D view; checkCIF report

## Figures and Tables

**Table 1:** Hydrogen-bond geometry (Å, °)

*D*—H⋯*A*	*D*—H	H⋯*A*	*D*⋯*A*	*D*—H⋯*A*
N1—H11⋯O1	0.88 (1)	2.09 (2)	2.866 (7)	146 (2)
N1—H12⋯O2^i^	0.87 (1)	2.21 (2)	3.040 (5)	160 (3)
N1—H12⋯O2′^i^	0.87 (1)	2.21 (2)	3.009 (5)	152 (3)
N2—H21⋯O5^ii^	0.88 (1)	2.26 (1)	3.075 (2)	155 (2)
N2—H22⋯O6^iii^	0.88 (1)	2.22 (2)	3.035 (2)	154 (2)
N3—H31⋯O4^iv^	0.88 (1)	2.28 (1)	3.140 (2)	166 (2)
N3—H32⋯O4^iii^	0.88 (1)	2.34 (1)	3.210 (2)	169 (2)
N5—H51⋯O4^iv^	0.88 (1)	2.25 (1)	3.093 (3)	159 (2)
N5—H52⋯O3^v^	0.88 (1)	2.10 (2)	2.860 (6)	144 (2)
N5—H52⋯O3′^v^	0.88 (1)	2.20 (2)	3.016 (6)	155 (2)
N6—H61⋯O1	0.88 (1)	2.02 (2)	2.88 (1)	168 (2)
N6—H62⋯O6^iii^	0.88 (1)	2.26 (2)	3.055 (2)	151 (2)

Symmetry codes: (i) 
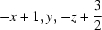
; (ii) 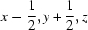; (iii) 
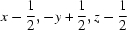
; (iv) 
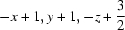
; (v) 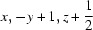.

**Fig. 1.**
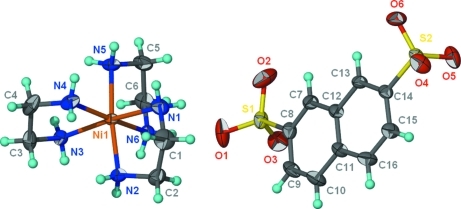
A displacement ellipsoid plot of [Ni(en)3] (C10H6O6S2) at the 50% probability level; hydrogen atoms are drawn as spheres of arbitrary radius; the disorder in the –SO3 is not shown.

## supplementary crystallographic information

### Comment

A previous study reported the crystal structure of tris(ethylenediamine)nickel(II) 2,6-naphthalenedisulfonate, which exists as a monohydrated salt (Huo *et al.*, 2004). The present 2,7-napthalenedisulfonate is an anhydrous salt (Scheme I). The Ni^II^ atom in Ni(en)_3_]^2+^ (C_10_H_6_O_6_S_2_)^2-^ is chelated by the en ligands in an octahedral geometry (Fig. 1). The cation and anion are linked by N–H···O hydrogen bonds into a hydrogen-bonded three-dimensional network (Table 1). One of the two –SO_3_ groups is disordered over two positions in a 1:1 ratio.

### Experimental

Nickel nitrate (1 mmol) and sodium 2,7-naphthalenedisulfonate (1 mmol) were dissolved in water (10 mol); the pH was adjusted to *ca* 6 by the dropwise addition of ethylenediamine. The solution was filtered; the solvent was allowed to evaporate for several days. Red crystals were isolated from the filtrate after several days.

### Refinement

C-bound H-atoms were generated geometrically and were included in the riding model approximation [C–H 0.93–0.97 Å, *U* 1.2*U*_eq_(C)]. The amino H atoms were located in a difference Fourier map, and were refined with a distance restraint of N–H 0.88±0.01 Å; their temperature factors were refined.

One sulfonate –SO_3_ group is disordered over two positions in respect of the O atoms. Each pair of S–O/S–O' distances were restrained to within 0.01 Å of each other, and the temperature factors of the primed atoms were set to those of the unprimed ones. The anisotropic temperature factors of the disordered atoms were restrained to be nearly isotropic. The occupancy could not be refined, and the disorder was assumed to be a 1:1 type of disorder.

### Crystal data

**Table:**

[Ni(C_2_H_8_N_2_)_3_](C_10_H_6_O_6_S_2_)	*F*(000) = 2208
*M**_r_* = 525.59	*D*_x_ = 1.561 Mg m^−^^3^
Monoclinic, *C*2/*c*	Mo *K*α radiation, λ = 0.71073 Å
Hall symbol: -C 2yc	Cell parameters from 18982 reflections
*a* = 23.624 (8) Å	θ = 3.1–27.5°
*b* = 14.203 (6) Å	µ = 1.10 mm^−^^1^
*c* = 14.715 (4) Å	*T* = 293 K
β = 115.152 (12)°	Prism, red
*V* = 4469 (3) Å^3^	0.20 × 0.16 × 0.13 mm
*Z* = 8	

### Data collection

**Table:**

Rigaku R-AXIS RAPID IP diffractometer	5107 independent reflections
Radiation source: fine-focus sealed tube	4533 reflections with *I* > 2σ(*I*)
graphite	*R*_int_ = 0.015
ω scan	θ_max_ = 27.5°, θ_min_ = 3.1°
Absorption correction: multi-scan (*ABSCOR*; Higashi, 1995)	*h* = −25→30
*T*_min_ = 0.810, *T*_max_ = 0.870	*k* = −18→18
21605 measured reflections	*l* = −19→17

### Refinement

**Table:**

Refinement on *F*^2^	Primary atom site location: structure-invariant direct methods
Least-squares matrix: full	Secondary atom site location: difference Fourier map
*R*[*F*^2^ > 2σ(*F*^2^)] = 0.031	Hydrogen site location: inferred from neighbouring sites
*wR*(*F*^2^) = 0.084	H atoms treated by a mixture of independent and constrained refinement
*S* = 1.05	*w* = 1/[σ^2^(*F*_o_^2^) + (0.0494*P*)^2^ + 3.411*P*] where *P* = (*F*_o_^2^ + 2*F*_c_^2^)/3
5107 reflections	(Δ/σ)_max_ = 0.001
337 parameters	Δρ_max_ = 0.57 e Å^−^^3^
36 restraints	Δρ_min_ = −0.36 e Å^−^^3^

### Fractional atomic coordinates and isotropic or equivalent isotropic displacement parameters (Å^2^)

**Table:**

	*x*	*y*	*z*	*U*_iso_*/*U*_eq_	Occ. (<1)
Ni1	0.323671 (9)	0.546855 (14)	0.654094 (14)	0.02520 (8)	
S1	0.45513 (2)	0.33944 (3)	0.52210 (3)	0.03989 (13)	
S2	0.672875 (18)	−0.08335 (3)	0.81143 (3)	0.02922 (10)	
O1	0.4030 (3)	0.3752 (9)	0.5376 (6)	0.0735 (17)	0.50
O2	0.5155 (2)	0.3831 (7)	0.5842 (5)	0.0750 (18)	0.50
O3	0.4427 (3)	0.3397 (11)	0.4172 (4)	0.0563 (13)	0.50
O1'	0.3896 (2)	0.3602 (9)	0.5021 (6)	0.0735 (17)	0.50
O2'	0.4995 (3)	0.3925 (7)	0.6050 (5)	0.0750 (18)	0.50
O3'	0.4620 (3)	0.3435 (11)	0.4303 (4)	0.0563 (13)	0.50
O4	0.66316 (7)	−0.16194 (11)	0.86591 (12)	0.0504 (4)	
O5	0.70273 (8)	−0.11148 (13)	0.74792 (12)	0.0570 (4)	
O6	0.70517 (7)	−0.00567 (11)	0.87655 (11)	0.0523 (4)	
N1	0.38181 (8)	0.42594 (13)	0.70904 (14)	0.0435 (4)	
H11	0.4042 (10)	0.4165 (18)	0.6747 (16)	0.049 (7)*	
H12	0.4088 (11)	0.428 (2)	0.7715 (9)	0.067 (8)*	
N2	0.25305 (7)	0.44232 (11)	0.59354 (12)	0.0337 (3)	
H21	0.2294 (9)	0.4393 (16)	0.6260 (15)	0.041 (6)*	
H22	0.2278 (10)	0.4548 (17)	0.5311 (9)	0.056 (7)*	
N3	0.25470 (7)	0.65642 (11)	0.60117 (12)	0.0332 (3)	
H31	0.2714 (11)	0.7125 (10)	0.6054 (18)	0.047 (6)*	
H32	0.2277 (9)	0.6498 (17)	0.5379 (9)	0.048 (6)*	
N4	0.30898 (9)	0.56411 (13)	0.78573 (12)	0.0409 (4)	
H41	0.2898 (10)	0.5151 (11)	0.7960 (17)	0.045 (6)*	
H42	0.3436 (8)	0.5686 (18)	0.8409 (13)	0.056 (7)*	
N5	0.39861 (8)	0.64335 (13)	0.70433 (12)	0.0390 (4)	
H51	0.3845 (11)	0.7013 (9)	0.7016 (19)	0.053 (7)*	
H52	0.4259 (9)	0.6361 (17)	0.7672 (9)	0.051 (7)*	
N6	0.33957 (7)	0.55196 (11)	0.52267 (11)	0.0327 (3)	
H61	0.3570 (10)	0.4988 (10)	0.5174 (17)	0.044 (6)*	
H62	0.3041 (7)	0.5556 (16)	0.4689 (12)	0.047 (7)*	
C1	0.34060 (12)	0.34445 (16)	0.6965 (2)	0.0574 (6)	
H1A	0.3276	0.3429	0.7508	0.069*	
H1B	0.3630	0.2867	0.6987	0.069*	
C2	0.28382 (10)	0.35180 (14)	0.59713 (17)	0.0448 (5)	
H2A	0.2963	0.3478	0.5424	0.054*	
H2B	0.2552	0.3005	0.5903	0.054*	
C3	0.22305 (11)	0.65778 (16)	0.66824 (17)	0.0459 (5)	
H3A	0.1939	0.6057	0.6524	0.055*	
H3B	0.1998	0.7160	0.6594	0.055*	
C4	0.27174 (12)	0.64973 (16)	0.77527 (16)	0.0515 (6)	
H4A	0.2988	0.7046	0.7927	0.062*	
H4B	0.2515	0.6468	0.8204	0.062*	
C5	0.42990 (9)	0.63874 (17)	0.63685 (16)	0.0474 (5)	
H5A	0.4568	0.5838	0.6524	0.057*	
H5B	0.4555	0.6944	0.6452	0.057*	
C6	0.38004 (10)	0.63295 (17)	0.53008 (15)	0.0454 (5)	
H6A	0.3555	0.6904	0.5126	0.054*	
H6B	0.3993	0.6252	0.4840	0.054*	
C7	0.52052 (9)	0.18670 (14)	0.62530 (14)	0.0380 (4)	
H7	0.5533	0.2288	0.6565	0.046*	
C8	0.46370 (9)	0.21874 (14)	0.55760 (14)	0.0377 (4)	
C9	0.41316 (9)	0.15619 (16)	0.51095 (16)	0.0464 (5)	
H9	0.3745	0.1791	0.4660	0.056*	
C10	0.42092 (9)	0.06234 (16)	0.53159 (17)	0.0479 (5)	
H10	0.3872	0.0217	0.5008	0.057*	
C11	0.47987 (8)	0.02544 (14)	0.59972 (14)	0.0363 (4)	
C12	0.52960 (8)	0.08928 (13)	0.64802 (13)	0.0335 (4)	
C13	0.58896 (8)	0.05348 (12)	0.71579 (13)	0.0328 (4)	
H13	0.6219	0.0946	0.7497	0.039*	
C14	0.59740 (8)	−0.04064 (12)	0.73078 (13)	0.0302 (3)	
C15	0.54825 (9)	−0.10479 (14)	0.68024 (15)	0.0397 (4)	
H15	0.5552	−0.1692	0.6899	0.048*	
C16	0.49068 (9)	−0.07183 (15)	0.61726 (16)	0.0431 (5)	
H16	0.4581	−0.1141	0.5855	0.052*	

### Atomic displacement parameters (Å^2^)

**Table:**

	*U*^11^	*U*^22^	*U*^33^	*U*^12^	*U*^13^	*U*^23^
Ni1	0.02437 (12)	0.02816 (12)	0.01999 (12)	−0.00189 (8)	0.00647 (8)	−0.00058 (7)
S1	0.0457 (3)	0.0396 (3)	0.0287 (2)	0.0178 (2)	0.01033 (19)	0.00466 (17)
S2	0.02485 (19)	0.0306 (2)	0.0274 (2)	0.00450 (16)	0.00644 (15)	0.00552 (15)
O1	0.084 (2)	0.062 (3)	0.098 (5)	0.038 (3)	0.061 (3)	0.016 (4)
O2	0.098 (3)	0.0375 (18)	0.045 (3)	0.012 (2)	−0.012 (2)	−0.0056 (16)
O3	0.063 (4)	0.0654 (16)	0.0404 (14)	0.001 (4)	0.022 (2)	0.0078 (18)
O1'	0.084 (2)	0.062 (3)	0.098 (5)	0.038 (3)	0.061 (3)	0.016 (4)
O2'	0.098 (3)	0.0375 (18)	0.045 (3)	0.012 (2)	−0.012 (2)	−0.0056 (16)
O3'	0.063 (4)	0.0654 (16)	0.0404 (14)	0.001 (4)	0.022 (2)	0.0078 (18)
O4	0.0445 (8)	0.0465 (9)	0.0551 (9)	0.0062 (6)	0.0162 (7)	0.0245 (7)
O5	0.0484 (9)	0.0748 (12)	0.0563 (10)	0.0202 (8)	0.0303 (8)	0.0078 (8)
O6	0.0402 (7)	0.0441 (8)	0.0463 (8)	0.0031 (6)	−0.0068 (6)	−0.0035 (6)
N1	0.0370 (9)	0.0459 (10)	0.0372 (9)	0.0073 (7)	0.0057 (7)	0.0097 (7)
N2	0.0318 (8)	0.0357 (8)	0.0309 (8)	−0.0049 (6)	0.0107 (6)	−0.0023 (6)
N3	0.0350 (8)	0.0330 (8)	0.0323 (8)	0.0006 (6)	0.0149 (6)	−0.0003 (6)
N4	0.0529 (10)	0.0448 (9)	0.0250 (8)	−0.0103 (8)	0.0166 (7)	−0.0026 (6)
N5	0.0314 (8)	0.0458 (10)	0.0324 (8)	−0.0097 (7)	0.0066 (6)	−0.0051 (7)
N6	0.0291 (7)	0.0410 (9)	0.0254 (7)	0.0042 (6)	0.0091 (6)	−0.0015 (6)
C1	0.0611 (14)	0.0381 (12)	0.0638 (15)	0.0082 (10)	0.0177 (12)	0.0202 (10)
C2	0.0517 (12)	0.0285 (9)	0.0530 (12)	−0.0058 (8)	0.0210 (10)	−0.0038 (8)
C3	0.0516 (12)	0.0439 (11)	0.0555 (13)	0.0064 (9)	0.0356 (10)	−0.0023 (9)
C4	0.0795 (16)	0.0455 (12)	0.0449 (12)	−0.0080 (11)	0.0412 (12)	−0.0122 (9)
C5	0.0323 (9)	0.0603 (13)	0.0495 (12)	−0.0126 (9)	0.0173 (9)	−0.0034 (10)
C6	0.0452 (11)	0.0566 (13)	0.0386 (11)	−0.0042 (9)	0.0219 (9)	0.0061 (9)
C7	0.0341 (9)	0.0348 (9)	0.0341 (9)	0.0053 (7)	0.0039 (7)	−0.0016 (7)
C8	0.0406 (10)	0.0390 (10)	0.0307 (9)	0.0128 (8)	0.0125 (8)	0.0041 (7)
C9	0.0274 (9)	0.0583 (13)	0.0431 (11)	0.0108 (8)	0.0050 (8)	0.0081 (9)
C10	0.0254 (9)	0.0552 (13)	0.0515 (12)	0.0008 (8)	0.0054 (8)	0.0072 (10)
C11	0.0257 (8)	0.0446 (10)	0.0350 (9)	0.0021 (7)	0.0094 (7)	0.0048 (7)
C12	0.0295 (8)	0.0356 (9)	0.0292 (8)	0.0050 (7)	0.0065 (7)	0.0016 (7)
C13	0.0271 (8)	0.0318 (9)	0.0301 (9)	0.0009 (6)	0.0032 (7)	−0.0001 (6)
C14	0.0260 (8)	0.0352 (9)	0.0260 (8)	0.0037 (6)	0.0078 (6)	0.0033 (6)
C15	0.0363 (9)	0.0325 (9)	0.0434 (10)	−0.0007 (7)	0.0104 (8)	0.0058 (7)
C16	0.0316 (9)	0.0418 (10)	0.0456 (11)	−0.0076 (8)	0.0065 (8)	0.0038 (8)

### Geometric parameters (Å, °)

**Table:**

Ni1—N5	2.1086 (17)	N6—H62	0.876 (10)
Ni1—N6	2.1222 (16)	C1—C2	1.510 (3)
Ni1—N4	2.1223 (17)	C1—H1A	0.9700
Ni1—N2	2.1255 (16)	C1—H1B	0.9700
Ni1—N1	2.1320 (19)	C2—H2A	0.9700
Ni1—N3	2.1457 (17)	C2—H2B	0.9700
S1—O3'	1.429 (5)	C3—C4	1.509 (3)
S1—O1	1.436 (5)	C3—H3A	0.9700
S1—O2'	1.438 (4)	C3—H3B	0.9700
S1—O3	1.444 (5)	C4—H4A	0.9700
S1—O2	1.465 (5)	C4—H4B	0.9700
S1—O1'	1.477 (5)	C5—C6	1.513 (3)
S1—C8	1.779 (2)	C5—H5A	0.9700
S2—O5	1.4459 (16)	C5—H5B	0.9700
S2—O6	1.4478 (16)	C6—H6A	0.9700
S2—O4	1.4484 (15)	C6—H6B	0.9700
S2—C14	1.7767 (18)	C7—C8	1.365 (3)
N1—C1	1.473 (3)	C7—C12	1.418 (3)
N1—H11	0.883 (10)	C7—H7	0.9300
N1—H12	0.869 (10)	C8—C9	1.410 (3)
N2—C2	1.467 (3)	C9—C10	1.362 (3)
N2—H21	0.877 (10)	C9—H9	0.9300
N2—H22	0.875 (10)	C10—C11	1.426 (3)
N3—C3	1.470 (2)	C10—H10	0.9300
N3—H31	0.879 (10)	C11—C16	1.409 (3)
N3—H32	0.882 (10)	C11—C12	1.413 (3)
N4—C4	1.470 (3)	C12—C13	1.425 (2)
N4—H41	0.878 (10)	C13—C14	1.356 (2)
N4—H42	0.876 (10)	C13—H13	0.9300
N5—C5	1.470 (3)	C14—C15	1.413 (3)
N5—H51	0.882 (10)	C15—C16	1.362 (3)
N5—H52	0.880 (10)	C15—H15	0.9300
N6—C6	1.470 (3)	C16—H16	0.9300
N6—H61	0.879 (10)		
			
N5—Ni1—N6	81.36 (7)	H61—N6—H62	106 (2)
N5—Ni1—N4	92.50 (7)	N1—C1—C2	109.55 (17)
N6—Ni1—N4	171.38 (7)	N1—C1—H1A	109.8
N5—Ni1—N2	173.40 (7)	C2—C1—H1A	109.8
N6—Ni1—N2	93.05 (6)	N1—C1—H1B	109.8
N4—Ni1—N2	93.42 (7)	C2—C1—H1B	109.8
N5—Ni1—N1	94.65 (8)	H1A—C1—H1B	108.2
N6—Ni1—N1	92.18 (7)	N2—C2—C1	108.54 (17)
N4—Ni1—N1	94.35 (7)	N2—C2—H2A	110.0
N2—Ni1—N1	81.99 (7)	C1—C2—H2A	110.0
N5—Ni1—N3	92.91 (7)	N2—C2—H2B	110.0
N6—Ni1—N3	92.62 (6)	C1—C2—H2B	110.0
N4—Ni1—N3	81.60 (7)	H2A—C2—H2B	108.4
N2—Ni1—N3	90.85 (7)	N3—C3—C4	108.66 (18)
N1—Ni1—N3	171.58 (7)	N3—C3—H3A	110.0
O3'—S1—O2'	116.5 (5)	C4—C3—H3A	110.0
O1—S1—O3	111.7 (4)	N3—C3—H3B	110.0
O1—S1—O2	115.1 (4)	C4—C3—H3B	110.0
O3—S1—O2	111.6 (4)	H3A—C3—H3B	108.3
O3'—S1—O1'	109.5 (4)	N4—C4—C3	109.20 (16)
O2'—S1—O1'	112.9 (4)	N4—C4—H4A	109.8
O3'—S1—C8	105.8 (6)	C3—C4—H4A	109.8
O1—S1—C8	107.0 (5)	N4—C4—H4B	109.8
O2'—S1—C8	107.8 (4)	C3—C4—H4B	109.8
O3—S1—C8	105.2 (6)	H4A—C4—H4B	108.3
O2—S1—C8	105.4 (4)	N5—C5—C6	108.11 (16)
O1'—S1—C8	103.3 (5)	N5—C5—H5A	110.1
O5—S2—O6	111.95 (11)	C6—C5—H5A	110.1
O5—S2—O4	112.36 (11)	N5—C5—H5B	110.1
O6—S2—O4	112.57 (10)	C6—C5—H5B	110.1
O5—S2—C14	106.82 (9)	H5A—C5—H5B	108.4
O6—S2—C14	106.19 (9)	N6—C6—C5	108.32 (16)
O4—S2—C14	106.42 (9)	N6—C6—H6A	110.0
C1—N1—Ni1	107.49 (13)	C5—C6—H6A	110.0
C1—N1—H11	109.5 (17)	N6—C6—H6B	110.0
Ni1—N1—H11	110.6 (17)	C5—C6—H6B	110.0
C1—N1—H12	108 (2)	H6A—C6—H6B	108.4
Ni1—N1—H12	116 (2)	C8—C7—C12	120.03 (18)
H11—N1—H12	105 (2)	C8—C7—H7	120.0
C2—N2—Ni1	107.97 (12)	C12—C7—H7	120.0
C2—N2—H21	111.0 (15)	C7—C8—C9	120.76 (18)
Ni1—N2—H21	111.9 (15)	C7—C8—S1	119.30 (16)
C2—N2—H22	108.7 (17)	C9—C8—S1	119.81 (14)
Ni1—N2—H22	111.2 (17)	C10—C9—C8	120.13 (18)
H21—N2—H22	106 (2)	C10—C9—H9	119.9
C3—N3—Ni1	106.88 (12)	C8—C9—H9	119.9
C3—N3—H31	106.3 (16)	C9—C10—C11	121.0 (2)
Ni1—N3—H31	112.5 (16)	C9—C10—H10	119.5
C3—N3—H32	111.3 (15)	C11—C10—H10	119.5
Ni1—N3—H32	113.8 (16)	C16—C11—C12	119.33 (17)
H31—N3—H32	106 (2)	C16—C11—C10	122.43 (18)
C4—N4—Ni1	108.82 (12)	C12—C11—C10	118.20 (19)
C4—N4—H41	110.1 (16)	C11—C12—C7	119.82 (16)
Ni1—N4—H41	111.3 (15)	C11—C12—C13	118.91 (17)
C4—N4—H42	108.5 (18)	C7—C12—C13	121.22 (17)
Ni1—N4—H42	113.8 (18)	C14—C13—C12	119.90 (17)
H41—N4—H42	104 (2)	C14—C13—H13	120.0
C5—N5—Ni1	108.82 (12)	C12—C13—H13	120.0
C5—N5—H51	107.5 (17)	C13—C14—C15	121.26 (16)
Ni1—N5—H51	110.2 (17)	C13—C14—S2	118.80 (14)
C5—N5—H52	110.6 (16)	C15—C14—S2	119.88 (14)
Ni1—N5—H52	115.0 (16)	C16—C15—C14	119.72 (18)
H51—N5—H52	104 (2)	C16—C15—H15	120.1
C6—N6—Ni1	108.70 (12)	C14—C15—H15	120.1
C6—N6—H61	111.4 (15)	C15—C16—C11	120.82 (18)
Ni1—N6—H61	108.8 (15)	C15—C16—H16	119.6
C6—N6—H62	111.4 (15)	C11—C16—H16	119.6
Ni1—N6—H62	110.6 (16)		
			
N5—Ni1—N1—C1	172.34 (16)	O2'—S1—C8—C7	−28.5 (3)
N6—Ni1—N1—C1	−106.15 (16)	O3—S1—C8—C7	114.1 (3)
N4—Ni1—N1—C1	79.47 (16)	O2—S1—C8—C7	−4.0 (3)
N2—Ni1—N1—C1	−13.37 (15)	O1'—S1—C8—C7	−148.2 (3)
N6—Ni1—N2—C2	75.95 (13)	O3'—S1—C8—C9	−79.1 (3)
N4—Ni1—N2—C2	−109.74 (13)	O1—S1—C8—C9	57.1 (4)
N1—Ni1—N2—C2	−15.83 (13)	O2'—S1—C8—C9	155.6 (3)
N3—Ni1—N2—C2	168.62 (13)	O3—S1—C8—C9	−61.8 (3)
N5—Ni1—N3—C3	−110.34 (13)	O2—S1—C8—C9	−179.9 (3)
N6—Ni1—N3—C3	168.18 (13)	O1'—S1—C8—C9	35.9 (3)
N4—Ni1—N3—C3	−18.25 (13)	C7—C8—C9—C10	−1.3 (3)
N2—Ni1—N3—C3	75.08 (13)	S1—C8—C9—C10	174.52 (18)
N5—Ni1—N4—C4	81.54 (15)	C8—C9—C10—C11	−0.4 (4)
N2—Ni1—N4—C4	−101.39 (15)	C9—C10—C11—C16	−175.4 (2)
N1—Ni1—N4—C4	176.40 (14)	C9—C10—C11—C12	2.1 (3)
N3—Ni1—N4—C4	−11.03 (14)	C16—C11—C12—C7	175.37 (19)
N6—Ni1—N5—C5	−15.85 (14)	C10—C11—C12—C7	−2.2 (3)
N4—Ni1—N5—C5	170.23 (15)	C16—C11—C12—C13	−2.0 (3)
N1—Ni1—N5—C5	75.66 (15)	C10—C11—C12—C13	−179.54 (19)
N3—Ni1—N5—C5	−108.06 (14)	C8—C7—C12—C11	0.6 (3)
N5—Ni1—N6—C6	−13.86 (13)	C8—C7—C12—C13	177.86 (18)
N2—Ni1—N6—C6	169.68 (13)	C11—C12—C13—C14	1.8 (3)
N1—Ni1—N6—C6	−108.23 (14)	C7—C12—C13—C14	−175.46 (18)
N3—Ni1—N6—C6	78.68 (13)	C12—C13—C14—C15	0.1 (3)
Ni1—N1—C1—C2	40.1 (2)	C12—C13—C14—S2	177.22 (14)
Ni1—N2—C2—C1	41.9 (2)	O5—S2—C14—C13	−97.28 (17)
N1—C1—C2—N2	−56.0 (3)	O6—S2—C14—C13	22.35 (18)
Ni1—N3—C3—C4	44.09 (18)	O4—S2—C14—C13	142.50 (16)
Ni1—N4—C4—C3	38.2 (2)	O5—S2—C14—C15	79.92 (17)
N3—C3—C4—N4	−56.2 (2)	O6—S2—C14—C15	−160.44 (16)
Ni1—N5—C5—C6	42.2 (2)	O4—S2—C14—C15	−40.30 (18)
Ni1—N6—C6—C5	40.48 (19)	C13—C14—C15—C16	−1.9 (3)
N5—C5—C6—N6	−55.5 (2)	S2—C14—C15—C16	−179.00 (16)
C12—C7—C8—C9	1.2 (3)	C14—C15—C16—C11	1.7 (3)
C12—C7—C8—S1	−174.65 (15)	C12—C11—C16—C15	0.2 (3)
O3'—S1—C8—C7	96.8 (3)	C10—C11—C16—C15	177.7 (2)
O1—S1—C8—C7	−127.0 (3)		

### Hydrogen-bond geometry (Å, °)

**Table:**

*D*—H···*A*	*D*—H	H···*A*	*D*···*A*	*D*—H···*A*
N1—H11···O1	0.88 (1)	2.09 (2)	2.866 (7)	146 (2)
N1—H12···O2^i^	0.87 (1)	2.21 (2)	3.040 (5)	160 (3)
N1—H12···O2'^i^	0.87 (1)	2.21 (2)	3.009 (5)	152 (3)
N2—H21···O5^ii^	0.88 (1)	2.26 (1)	3.075 (2)	155 (2)
N2—H22···O6^iii^	0.88 (1)	2.22 (2)	3.035 (2)	154 (2)
N3—H31···O4^iv^	0.88 (1)	2.28 (1)	3.140 (2)	166 (2)
N3—H32···O4^iii^	0.88 (1)	2.34 (1)	3.210 (2)	169 (2)
N5—H51···O4^iv^	0.88 (1)	2.25 (1)	3.093 (3)	159 (2)
N5—H52···O3^v^	0.88 (1)	2.10 (2)	2.860 (6)	144 (2)
N5—H52···O3'^v^	0.88 (1)	2.20 (2)	3.016 (6)	155 (2)
N6—H61···O1	0.88 (1)	2.02 (2)	2.88 (1)	168 (2)
N6—H62···O6^iii^	0.88 (1)	2.26 (2)	3.055 (2)	151 (2)

Symmetry codes: (i) −*x*+1, *y*, −*z*+3/2; (ii) *x*−1/2, *y*+1/2, *z*; (iii) *x*−1/2, −*y*+1/2, *z*−1/2; (iv) −*x*+1, *y*+1, −*z*+3/2; (v) *x*, −*y*+1, *z*+1/2.
